# Flavonoids-Rich Plant Extracts Against *Helicobacter pylori* Infection as Prevention to Gastric Cancer

**DOI:** 10.3389/fphar.2022.951125

**Published:** 2022-08-31

**Authors:** Renaly Ivyna de Araújo Rêgo, Geovana Ferreira Guedes Silvestre, Demis Ferreira de Melo, Sonaly Lima Albino, Marcela Monteiro Pimentel, Sara Brito Silva Costa Cruz, Sabrina Daniela Silva Wurzba, Wellington Francisco Rodrigues, Bolívar Ponciano Goulart de Lima Damasceno, Lúcio Roberto Cançado Castellano

**Affiliations:** ^1^ Human Immunology Research and Education Group-GEPIH, Federal University of Paraiba, João Pessoa, Brazil; ^2^ Postgraduate Program of Pharmaceutical Sciences, State University of Paraíba, Campina Grande, Brazil; ^3^ Postgraduate Program of Science and Technology in Health, State University of Paraíba, Campina Grande, Brazil; ^4^ Postgraduate Program of Therapeutic Innovation, Federal University of Pernambuco, Recife, Brazil; ^5^ Postgraduate Program in Dentistry, Federal University of Paraíba, João Pessoa, Brazil; ^6^ Department of Otolaryngology and Head and Neck Surgery, McGill University, Montreal, QC, Canada; ^7^ Segal Cancer Centre and Lady Davis Institute for Medical Research, Departments of Medicine and Oncology, Faculty of Medicine, McGill University, Montreal, QC, Canada; ^8^ Postgraduate Course in Health Sciences, Federal University of Triangulo Mineiro, UFTM, Uberaba, Brazil

**Keywords:** phytotherapy, stomach neoplasms, anti-infective agents, ethnopharmacology, preventive medicine

## Abstract

Gastric cancer is the fifth most common and fourth type to cause the highest mortality rates worldwide. The leading cause is related to *Helicobacter pylori* (*H. pylori*) infection. Unfortunately, current treatments have low success rates, highlighting the need for alternative treatments against carcinogenic agents, specifically *H. pylori*. Noteworthy, natural origin products contain pharmacologically active metabolites such as flavonoids, with potential antimicrobial applications.

**Objective:** This article overviews flavonoid-rich extracts’ biological and pharmacological activities. It focuses on using these substances against *Helicobacter pylori* infection to prevent gastric cancer. For this, PubMed and Science Direct databases were searched for studies that reported the activity of flavonoids against *H. pylori*, published within a 10-year time frame (2010 to August 2020). It resulted in 1,773 publications, of which 44 were selected according to the search criteria. The plant family primarily found in publications was Fabaceae (9.61%). Among the flavonoids identified after extraction, the most prevalent were quercetin (19.61%), catechin (13.72), epicatechin (11.76), and rutin (11.76). The potential mechanisms associated with anti-*H. pylori* activity to the extracts were: inhibition of urease, damage to genetic material, inhibition of protein synthesis, and adhesion of the microorganism to host cells.

**Conclusion:** Plant extracts rich in flavonoids with anti-*H. pylori* potential proved to be a promising alternative therapy source, reinforcing the relevance of studies with natural products.

## Graphical Abstract

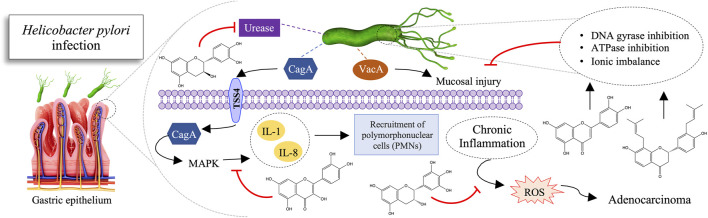

## Introduction

Gastric cancer represents the fifth most common type of cancer (1.08 million cases in 2020). It is the fourth most common cause of death among cancers worldwide (768.000 deaths in 2020) due to the advanced stage at diagnosis (Smyth et al., 2020; Sung et al., 2021). Incidence and mortality vary considerably between regions, although it is more prevalent in developing countries, in which the percentage of cases is equivalent to 70%, especially in East Asia. In addition, the incidence of gastric cancer is proportional to population age, with an average of 68 years, and is more common in men (1 in each 96) than in women (1 in each 152) (Herrero et al., 2014; medical and editorial content Team, 2018).

According to the topology, gastric cancer can be classified into cardia, usually associated with gastroesophageal reflux, and non-cardia or distal gastric cancer, caused by the interaction with different factors (Nardone, 2006; de Martel et al., 2013). The latter is histologically subdivided according to Laurén’s classification in diffuse and intestinal (Lauren, 1965). The diffuse type consists of individually infiltrated neoplastic cells without glandular structures. In contrast, the intestinal type mimics the intestinal glands. It progresses through a series of histological changes that begin with transitioning from normal mucosa to chronic superficial gastritis, followed by atrophic gastritis, intestinal metaplasia, dysplasia, and adenocarcinoma (Nardone, 2006; Polk and Peek, 2010). For both types of non-cardiac gastric cancer, the main factor associated with developing approximately 90% of adenocarcinomas is the bacterium *Helicobacter pylori* (*H. pylori*) (Peek, 2005; Plummer et al., 2015, 2016; Moss, 2017; Thrift and El-Serag, 2020).

*H. pylori* is a gram-negative, flagellated, microaerophilic bacteria that infects the epithelial lining of the stomach (Tsukamoto and Tatematsu, 2014; Hooi et al., 2017). The infection is prevalent in approximately 50% of the world population, varying according to geographic region, age, socioeconomic status, education level, environment, and occupation. It is usually contracted in the first years of life and tends to persist until the completion of the appropriate treatment (McColl, 2010; Cui et al., 2013; Wang et al., 2014; Schaalan et al., 2020).

The inflammatory process developed by *H. pylori* infection involves a variety of pathways induced in both gastric epithelial cells and circulating immune cells recruited to the infection site. Activated pathways involve mitogen-activated protein kinase (MAPK), nuclear factor-κB (NF-kB), activating protein (AP)-1, Wnt/β-catenin, PI3K pathways, signal transducers and transcription activators 3 (STAT3). These alterations cause an increase in the production of inflammatory cytokines, such as interleukin 1 (IL-1), IL-6, IL-8, and tumor necrosis factor-alpha (TNF-α). Also, they alter the apoptosis rate and proliferation and differentiation of epithelial cells. These phenomena result in the oncogenic transformation of epithelial cells and gastric cancer formation (Ding et al., 2010; Lamb and Chen, 2013; Schaalan et al., 2020). In addition, virulence factors contribute to determining the pattern of immune defense performed in response to the infection. It includes the factors named vacuolating cytotoxin (VacA), cytotoxin-associated antigen A (CagA), the Cag pathogenicity island (PAI), HP-NAP, oipA, and dupA (Ding et al., 2010; Sepulveda, 2013; Wang et al., 2014; Yamaoka, 2010, 2012).

The clinical condition produced by the microorganism in question is usually asymptomatic. However, the infection caused is associated with gastrointestinal diseases, such as chronic gastritis, peptic ulcer disease, gastric B-cell mucosa-associated lymphoid tissue lymphoma, and, as aforementioned, gastric adenocarcinoma. Thereby, *H. pylori* was recognized and classified as a definite (group 1) carcinogen by the World Health Organization’s International Agency for Research on Cancer in 1994 (WHO, 2010; de Martel et al., 2013; Plummer et al., 2016; Moss, 2017). Additionally, studies demonstrate that eradicating *H. pylori* decreases the risk of developing cancer in individuals without pre-malignant lesions. It reinforces that this infection influences the early stages of gastric carcinogenesis (Polk and Peek, 2010).

Currently, the treatment for gastric cancer consists of surgical intervention associated with chemotherapy using 5-fluorouracil (5-FU), platinum, taxane, irinotecan, and anthracycline (Ma et al., 2016). The treatment options for tumors associated with *H. pylori* are antibiotics (clarithromycin and amoxicillin or metronidazole), proton pump inhibitors, and bismuth (Lamb and Chen, 2013). However, surgery remains the only curative therapy. At the same time, chemotherapy can improve the outcome, thus emphasizing the importance of employing preventive recourses (Orditura et al., 2014).

Alternatively, there is an increase in the use of natural products. One of the most investigated sources is the plants, representing 25% of the medical industry (Ji et al., 2009; Lahlou, 2013; Calixto, 2019). Botanical drugs contain active metabolites with pharmacological activities capable of relieving symptoms or curing diseases (Trojan-Rodrigues et al., 2012; Ferreira et al., 2014). Thus, the application of ethnopharmacology has collaborated in discovering new chemical entities, mainly through the bioprospecting of secondary metabolites (Albino et al., 2020).

Among the most known and studied ethnobotanical constituents are flavonoids. They possess a polyphenolic benzo- γ -pyrone structure of low molecular weight (Kumar and Pandey, 2013; Jucá et al., 2020). Moreover, these compounds present antioxidant (Babiaka et al., 2020; Joseph Sahayarayan et al., 2020; Liu et al., 2020), hepatoprotective (Ge et al., 2018; Ma Q. et al., 2020; Wei et al., 2020), anti-inflammatory (Maleki et al., 2019; Tian et al., 2019; Wu et al., 2019), anticancer (Imran et al., 2019; Bailly, 2020; Wei et al., 2020), antiviral (Ahmad et al., 2015; Russo et al., 2020), and antibacterial properties (Cushnie and Lamb, 2005; Ahmad et al., 2015; Tian et al., 2019; Biharee et al., 2020). Concerning the antibacterial activity, flavonoids can inhibit the synthesis of nucleic acids, the function of the cytoplasmic membrane, and energy metabolism, among others. These activities prompt their application as antibacterial drugs with a scope of possible mechanisms of action (Cushnie and Lamb, 2005; Xie et al., 2014).

The antimicrobial potential of flavonoids against *H. pylori* has been described in the literature by several studies (Loo, 1997; Bae et al., 1999; Shin et al., 2005; Lee et al., 2006; Cushnie and Lamb, 2011; Pandey and Kumar, 2013; González et al., 2019). In addition to demonstrating an *in vitro* action against this bacterium, these compounds could also promote synergistic interactions with antibiotics commonly used in treatments against *H. pylori* infections (Krzyżek et al., 2021). The pathways of action of flavonoids can be diverse. Some mechanisms in *H. pylori* have already been described, such as inhibition of the essential function of HsrA (González et al., 2019), mediation of the response to oxidative stress (Olekhnovich et al., 2013, 2014; Pelliciari et al., 2017), interactions with virulence factors (Kim et al., 2021), recognition of molecular targets including secretion systems (Yeon et al., 2019) and enzymes (Wu et al., 2008; Zhang et al., 2008) acting on pathways that lead to changes in cell morphology of *H. pylori* (Krzyżek et al., 2021). In turn, the broad pharmacotherapeutic and biochemical spectrum of flavonoids and the possible contribution of these compounds to improving human health make such substances increasingly explored (Tungmunnithum et al., 2018). Still, these findings support the valuable potential of flavonoids as candidate botanical drugs for novel antibacterial and anticancer strategies. The most promising flavonoid compounds are Catechin, Epicatechin, Kaempferol, Luteolin, Morin, Myricetin, Naringenin, Naringin, Quercetin, Hyperoside, and Rutin, In this way, this article provides an overview of the biological and pharmacological activities of flavonoid-rich plant extracts with a focus on the use of these substances against *H. pylori* infection in the prevention of gastric cancer.

## Materials and Methods

### The Question Under Analysis

This review was guided by the question: “Are flavonoids-rich plant extracts a promising alternative in treatments against *Helicobacter pylori* infection and preventing gastric cancer?”

### Search Strategy and Articles Selection

PRISMA guidelines were followed (Liberati et al., 2009; Moher et al., 2009). In addition, an electronic search was performed in the PubMed and Science Direct databases from studies published between 2010 to August 2020, with the keywords “flavonoids” and “*Helicobacter pylori*.”

### Studies Selection

Eligible studies followed the criteria: *1*) pre-clinical *in vitro* and *in vivo*; *2*) studies with rodents and cells; *3*) any type of treatment that used plant extracts containing flavonoids in its composition; *4*) studies with positive or negative control; *5*) no language restriction. Clinical research, studies with other than flavonoids that did not determine the value of the tested dose, with flavonoids in their isolated form, and studies that used flavonoids as control compounds, were excluded

Two independent reviewers selected the studies. In the first screening, titles and abstracts were evaluated, and studies considered irrelevant were excluded. The two reviewers read the articles for each potential manuscript and evaluated them based on the inclusion criteria. Duplicate studies between the bases were excluded. A third reviewer was contacted in the presence of inconsistency between the two examiners. Thus, 44 articles were selected for this review that reports the activity of plant species extracts containing flavonoids against *H. pylori*.

## Results

**FIGURE 1:**
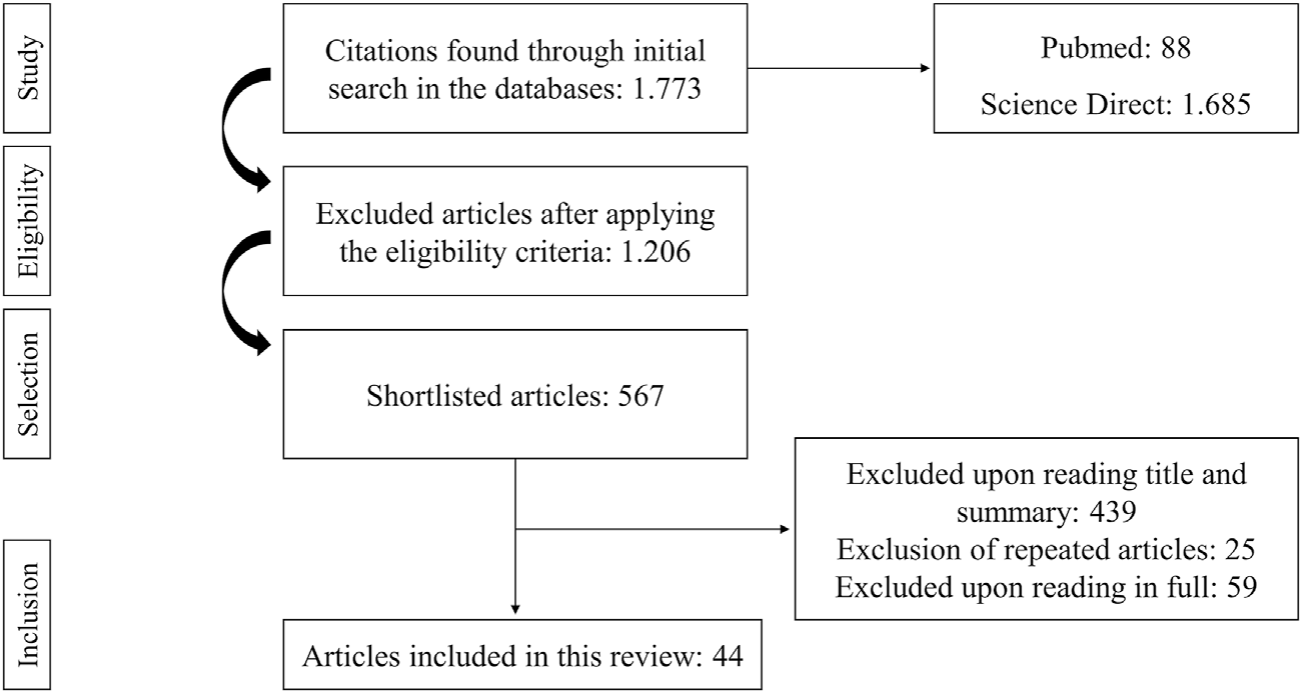
Flowchart of article selection for the systematic review. The bibliographic study started with 1.773 articles. After applying the eligibility criteria, 567 remained. Four hundred thirty-nine were excluded after reading the title, 25 excluded by repetition, and 59 excluded after full reading. Forty-four articles fit the purpose and were selected for this review.

The initial search of the databases (with the strategies presented in Table 1) allowed the identification of 1,773 publications. Review studies, meta-analyzes, encyclopedias, book chapters, abstracts, conference proceedings, editorials/letters, and case reports were excluded. The 567 articles left were screened based on titles and abstracts for the inclusion criteria mentioned above. At this stage, 128 articles remained, following the exclusion of 439 articles. Subsequently to the removal of 25 repeated articles, 103 remained. These studies were afterward entirely read. Finally, 44 articles were selected, as 59 articles did not meet all inclusion criteria (Figure 1). The selected studies were concentrated between 2010 and 2020 and are considered current.

**TABLE 1:** Pharmacobotanical information, extracts and tests involving activity against *H. pylori* of flavonoids contained in plant species.

Species	Family	Extracted part	Identified flavonoids	Study model	Pharmacological evaluation	Country	References
*Qualea parviflora*	Vochysiaceae	Bark	—	*In vitro*	Agar well diffusion	Brazil	Mazzolin et al. (2010)
*Camellia sinensis*	Theaceae	Leaves	Catechin; Epicatechin Epigallocatechin; Quercetin	*In vitro*	Agar diffusion	USA	Ankolekar et al. (2011)
*Hypericum erectum*	Hypericaceae	Whole plant	Quercetin-3′-O-β-D-galactopyranoside	*In vitro*	MIC	South Korea	Moon et al. (2011)
*Bridelia micranta*	Phyllanthaceae	Stem bark	—	*In vitro*	Agar well diffusion, MIC, rate of kill	South Africa	Okeleye et al. (2011)
*Byrsonima intermedi*	Malpighiaceae	Leaves	Catechin; Epicatechin; Quercetin Quercetin-3-(2″-O-galloyl)-O-α-galactopyranoside; Quercetin-3-O-(2″-O-galloyl)-α- arabinopyranoside; Quercetin-3′-O-(2″-acetyl)-β-D-glucopyranoside; Quercetin-3-O-α-arabinopyranoside; Quercetin-3′-O-β-D-galactopyranoside 7,3′-di-O- methyleriodictyol	*In vitro*	MIC	Brazil	Santos et al. (2012)
*Glycyrrhiza Glabra*	Fabaceae	Roots	Glabridin; Glabrol	*In vitro*	MIC	India	Asha et al. (2013)
*Amygdalus communis*	Rosaceae	Fruit	Epicatechin; Naringenin	*In vitro*	MIC	Italy	Bisignano et al. (2013)
*Vitis rotundifolia*	Vitaceae	Fruit	—	*In vitro*	Disk diffusion	USA	Brown and Jiang, (2013)
*Polygala cyparissias*	Polygalaceae	Whole plant	—	*In vitro*	MIC	Brazil	Klein-Júnior et al. (2013)
*Lythrum salicaria*	Lythraceae	Leaves, flowers and stem	—	*In vitro*	Disk diffusion	Iran	Manayi et al. (2013)
*Caesalpinia pyramidalis*	Leguminosae	Inner bark	—	*In vitro*	MIC, MBC	Brazil	Ribeiro et al. (2013)
*Hippocratea celastroides*	Hippocrateaceae	Leaves, stems, and root bark	—	*In vitro*	MIC	Mexico	Escobedo Hinojosa et al. (2014)
*Theobroma cacao*	Malvaceae	Seeds	—	*In vitro*	MIC	Nigeria	Lawal et al. (2014)
*Lippia integrifolia*	Lythraceae	Leaves and flowers	Salvagenin; 6-Hydroxyluteolin 7-hexoside; 6-Methoxyluteolin-hexoside 6-Methylscutellarein 7-hexoside B-ring-dimethoxylated Flavone- hexoside; Methoxylated apigenin-hexoside	*In vitro*	Agar diffusion	Argentina	Marcial et al. (2014)
*Cuphea aequipetala*	Lythraceae	Leaves and flowers	—	*In vitro*	MIC	Brazil	Palacios-Espinosa et al. (2014)
*Peumus boldus*	Monimiaceae	Leaves	Catechin; Epicatechin	*In vitro*	MIC	Chile	Pastene et al. (2014)
*Solanum cernuum*	Solanaceae	Leaves	Afzelin; Quercitrin	*In vitro*	MIC, MBC	Brazil	Miranda et al. (2015)
*Copaifera malmei*	Fabaceae	Leaves	Rutin; Catechin; Quercetin	*In vitro*	MIC	Brazil	Adzu et al. (2015)
*Parthenium hysterophorus*	Asteraceae	Roots	—	*In vitro*	MIC	Mexico	Espinosa-Rivero et al. (2015)
*Lithraea molleoides*	Anacardaceae	Leaves	Rutin	*In vitro*	MIC	Argentina	Garro et al. (2015)
*Syzygium aromaticum*; *Piper nigrum*; *Cuminum cyminum*; *Salvia officinalis*; *Punica granatum*; *Zingiber officinale*; *Commiphora myrrha*; *Glycyrrhiza glabra*	Myrtaceae; Piperaceae; Apiaceae; Lamiaceae; Punicaceae; Zingiberaceae; Burseraceae Fabaceae	Flowers; Fruit; Seeds; Leaves; Peel; Roots; Resin; Roots	Catechin	*In vitro*	MIC	Egypt	Hamad et al. (2015)
*Maytenus robusta*	Celastraceae	Leaves	—	*In vitro*	MIC	Brazil	Mota Da Silva et al. (2015)
*Leonotis nepetifolia*	Lamiaceae	Whole plant	Kaempferol; Morin; Myricetin; Naringin; Naringenin; Quercetin; Rutin	*In vitro*	MIC, MBC	Brazil	Oliveira et al. (2015)
*Piper umbellatum*	Piperaceae	Leaves	—	*In vitro*	MIC	Brazil	da Silva Junior et al. (2016)
*Euphorbia umbellata*	Euphorbiaceae	Bark	—	*In vitro*	Disk diffusion	Brazil	Minozzo et al. (2016)
*Rosa hybrida*	Rosaceae	Flowers	—	*In vitro*	MIC	Korea	Park et al. (2016)
*Agrimonia eupatoria*; *Fragaria vesca*	Rosaceae	Leaves and stems; flowers and fruit	—	*In vitro*	MIC	Portugal	Cardoso et al. (2018)
*Heterotheca inuloides*	Asteraceae	Leaves, stems and flowers	Quercetin; 7,3′-di-O- methyleriodictyol	*In vitro*	MIC	Mexico	Egas et al. (2018)
*Anoda cristata; Cnidoscolus aconitifolius; Crotalaria pumila*	Malvaceae; Euphorbiaceae; Fabaceae	Leaves	Acacetin; Diosmetin	*In vitro*	MIC	Mexico	Gomez-Chang et al. (2018)
*Desmostachya bipinnata*	Poaceae	Leaves and flowers	—	*In vitro*	MIC	Saudi Arabia	Ibrahim et al. (2018)
*Oryza sativa*	Poaceae	Grain	—	*In vitro*	Western blotting	South Korea	Kim et al. (2018)
*Ixeris chinensis*	Asteraceae	Whole plant	Kaempferol; Luteolin; Myricetin; Naringenin; Naringin; Rutin	*In vitro*	Disk diffusion	Taiwan	Lu et al. (2018)
*Physalis alkekengi*	Solanaceae	Leaves and flowers	Kaempferol; Quercetin	*In vitro*	MIC	China	Wang et al. (2018)
*Cannabis sativa*	Cannabaceae	Flowers	Catechin; Epicatechin; Naringenin; Naringin; Quercetin; Rutin	*In vitro*	MIC, MBC	Italy	Zengin et al. (2018)
*Cochlospermum regium*	Cochlospermaceae	Leaves	Kaempferol; Morin; Myricetin; Rutin	*In vitro*	MIC	Brazil	Arunachalam et al. (2019)
*Azadirachta indica*	Meliaceae	Fruit and seeds	—	*In vitro*	MIC	Italy	Cesa et al. (2019)
*Virola elongata*	Myristicaceae	Stems	—	*In vitro*	MIC	Brazil	de Almeida et al. (2019)
*Byrsonima intermedia*	Malpighiaceae	Leaves	Catechin; Epicatechin; Quercetin	*In vitro*	MIC	Brazil	de Cássia dos Santos et al. (2019)
*Diospyros virginiana*	Ebenaceae	Pedicels	—	*In vitro*	MIC	South Korea	Saravanakumar et al. (2019)
*Casearia sylvestris*	Salicaceae	Leaves	—	*In vitro*	MIC	Brazil	Spósito et al. (2019)
*Plectranthus barbatus*	Lamiaceae	Leaves	Luteolin; Quercetin	*In vitro*	MIC, MBC	Brazil	Borges et al. (2020)
*Berberis aristata*	Berberidaceae	Stems	—	*In vitro*	Disk diffusion	India	Das et al. (2020)
*Erythrina speciosa*	Fabaceae	Leaves	—	*In vitro*	MIC	Egypt	Fahmy et al. (2020)
*Alpinia Officinarum*	Zingiberaceae	Rhizomes	Apigenin; Galangin; Galangin-3-methylether; Kaempferol; Kaempferide; Pinobaksin; Ponocembrin; Quercetin; Quercetin-3-methylether; Salvagenin	*In vivo*	*H. pylori*-associated gastritis (HAG) model (mice)	China	Ma et al. (2020)

There was variability in the study regions for the selected manuscripts, with 25% of the papers coming from Asia (Moon et al., 2011; Asha et al., 2013; Manayi et al., 2013; Wang et al., 2014; Park et al., 2016; Ibrahim et al., 2018; Kim et al., 2018; Lu et al., 2018; Saravanakumar et al., 2019; Ma X. et al., 2020; Das et al., 2020), 56.82% from America, of which 40.91% were from South America (Adzu et al., 2015; Almeida et al., 2019; Arunachalam et al., 2019; Borges et al., 2020; da Silva Junior et al., 2016; de Cássia dos Santos et al., 2019; Garro et al., 2015; Klein-Júnior et al., 2013; Marcial et al., 2014; Mazzolin et al., 2010; Minozzo et al., 2016; Miranda et al., 2015; Mota Da Silva et al., 2015; Oliveira et al., 2015; Pastene et al., 2014; Ribeiro et al., 2013; Santos et al., 2012; Spósito et al., 2019) and 15.91% from North America (Ankolekar et al., 2011; Brown and Jiang, 2013; Escobedo Hinojosa et al., 2014; Palacios-Espinosa et al., 2014; Espinosa-Rivero et al., 2015; Egas et al., 2018; Gomez-Chang et al., 2018), 9.09% of the articles originated from the Africa (Okeleye et al., 2011; Lawal et al., 2014; Hamad et al., 2015; Fahmy et al., 2020), and 9,09% from the Europe (Bisignano et al., 2013; Cardoso et al., 2018; Zengin et al., 2018; Cesa et al., 2019).

Among the Asian countries, South Korea represented 9.09% of the publications (Moon et al., 2011; Park et al., 2016; Kim et al., 2018; Saravanakumar et al., 2019), India (Asha et al., 2013; Das et al., 2020) and China (Wang et al., 2018; Ma X. et al., 2020) both 4.55%, Iran (Manayi et al., 2013), Saudi Arabia (Ibrahim et al., 2018), and Taiwan (Lu et al., 2018) 2.27%. In South America, Brazil (Adzu et al., 2015; Almeida et al., 2019; Arunachalam et al., 2019; Borges et al., 2020; da Silva Junior et al., 2016; de Cássia dos Santos et al., 2019; Klein-Júnior et al., 2013; Mazzolin et al., 2010; Minozzo et al., 2016; Miranda et al., 2015; Mota Da Silva et al., 2015; Oliveira et al., 2015; Ribeiro et al., 2013; Santos et al., 2012; Spósito et al., 2019) represented 34.09%, Argentina (Marcial et al., 2014; Garro et al., 2015) 4.55%, and Chile (Pastene et al., 2014) 2.27%. In North America, Mexico (Escobedo Hinojosa et al., 2014; Palacios-Espinosa et al., 2014; Espinosa-Rivero et al., 2015; Egas et al., 2018; Gomez-Chang et al., 2018) represented 11.36% and the United States (Ankolekar et al., 2011; Brown and Jiang, 2013) 4.55%. In the Africa, Egypt (Hamad et al., 2015; Fahmy et al., 2020) represented 4.55%, Nigeria (Lawal et al., 2014) and South Africa (Okeleye et al., 2011) both 2.27%. In the Europe, Italy (Bisignano et al., 2013; Zengin et al., 2018; Cesa et al., 2019) represented 6.82% and Portugal (Cardoso et al., 2018) 2.27% of publications.

The plant families used in the studies were Anacardaceae (Garro et al., 2015), Apiaceae (Hamad et al., 2015), Asteraceae (Espinosa-Rivero et al., 2015; Egas et al., 2018; Lu et al., 2018), Berberidaceae (Das et al., 2020), Burseraceae (Hamad et al., 2015), Cannabaceae (Zengin et al., 2018), Celastraceae (Mota Da Silva et al., 2015), Cochlospermaceae (Arunachalam et al., 2019), Ebenaceae (Saravanakumar et al., 2019), Euphorbiaceae (Minozzo et al., 2016; Gomez-Chang et al., 2018), Fabaceae (Asha et al., 2013; Adzu et al., 2015; Hamad et al., 2015; Gomez-Chang et al., 2018; Fahmy et al., 2020), Hippocrateaceae (Escobedo Hinojosa et al., 2014), Hypericaceae (Moon et al., 2011), Lamiaceae (Oliveira et al., 2015), Leguminosae (Ribeiro et al., 2013), Lythraceae (Manayi et al., 2013; Marcial et al., 2014; Palacios-Espinosa et al., 2014), Malpighiaceae (Santos et al., 2012; de Cássia dos Santos et al., 2019), Malvaceae (Lawal et al., 2014; Gomez-Chang et al., 2018), Meliaceae (Cesa et al., 2019), Monimiaceae (Pastene et al., 2014), Myristicaceae (de Almeida et al., 2019), Myrtaceae (Hamad et al., 2015), Phyllanthaceae (Okeleye et al., 2011), Piperaceae (da Silva Junior et al., 2016; Hamad et al., 2015), Poaceae (Ibrahim et al., 2018; Kim et al., 2018), Polygalaceae (Klein-Júnior et al., 2013), Rosaceae (Bisignano et al., 2013; Park et al., 2016; Cardoso et al., 2018), Salicaceae (Spósito et al., 2019), Solanaceae (Abreu Miranda et al., 2015; Wang et al., 2018), Theaceae (Ankolekar et al., 2011), Vitaceae (Brown and Jiang, 2013), Vochysiaceae (Mazzolin et al., 2010) and Zingiberaceae (Hamad et al., 2015; Ma X. et al., 2020). The most prominent family in these studies was the Fabaceae.

Between the flavonoids identified in the extracts (Table 2), quercetin (Ankolekar et al., 2011; Santos et al., 2012; Adzu et al., 2015; Oliveira et al., 2015; Egas et al., 2018; Wang et al., 2018; Zengin et al., 2018; de Cássia dos Santos et al., 2019; Ma X. et al., 2020; Borges et al., 2020), catechin (Ankolekar et al., 2011; Santos et al., 2012; Pastene et al., 2014; Adzu et al., 2015; Hamad et al., 2015; Zengin et al., 2018; de Cássia dos Santos et al., 2019), epicatechin (Ankolekar et al., 2011; Santos et al., 2012; Bisignano et al., 2013; Pastene et al., 2014; Zengin et al., 2018; de Cássia dos Santos et al., 2019), rutin (Adzu et al., 2015; Garro et al., 2015; Oliveira et al., 2015; Lu et al., 2018; Zengin et al., 2018; Arunachalam et al., 2019), kaempferol (Oliveira et al., 2015; Lu et al., 2018; Wang et al., 2018; Arunachalam et al., 2019; Ma X. et al., 2020), naringenin (Bisignano et al., 2013; Oliveira et al., 2015; Lu et al., 2018; Zengin et al., 2018), naringin (Oliveira et al., 2015; Lu et al., 2018; Zengin et al., 2018), luteolin (Lu et al., 2018; Borges et al., 2020; Fahmy et al., 2020), myricetin (Oliveira et al., 2015; Lu et al., 2018; Arunachalam et al., 2019), morin (Oliveira et al., 2015; Arunachalam et al., 2019), and quercetin-3′-O-β-D-galactopyranoside (Moon et al., 2011; Santos et al., 2012) stood out.

**TABLE 2:** Main flavonoids identified in the articles included in this review.

ID	Name	Structure	Reference
01	Catechin	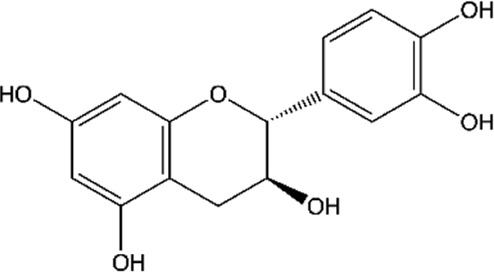	Adzu et al. (2015); Ankolekar et al. (2011); de Cássia dos Santos et al. (2019); Hamad et al. (2015); Pastene et al. (2014); Santos et al. (2012); Zengin et al. (2018)
02	Epicatechin	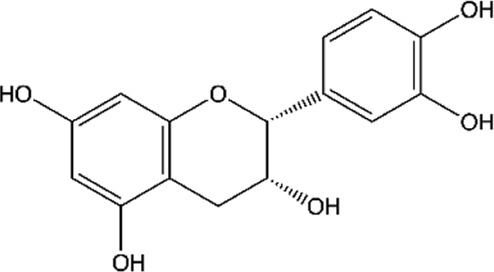	Ankolekar et al. (2011); Bisignano et al. (2013); de Cássia dos Santos et al. (2019); Pastene et al. (2014); Santos et al. (2012); Zengin et al. (2018)
03	Kaempferol	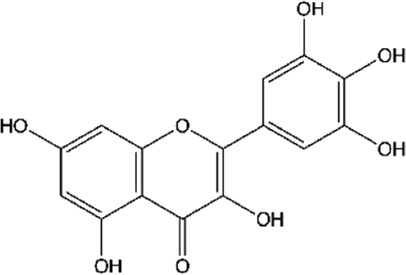	Arunachalam et al. (2019); Lu et al. (2018); Ma et al. (2020); Oliveira et al. (2015); Wang et al. (2018)
04	Luteolin	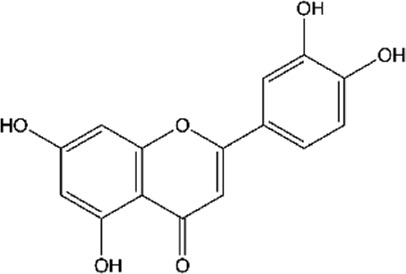	Borges et al. (2020); Fahmy et al. (2020); Lu et al. (2018)
05	Morin	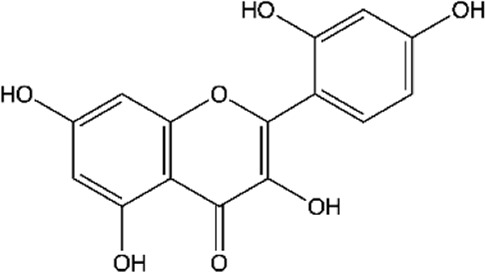	Arunachalam et al. (2019); Oliveira et al. (2015)
06	Myricetin	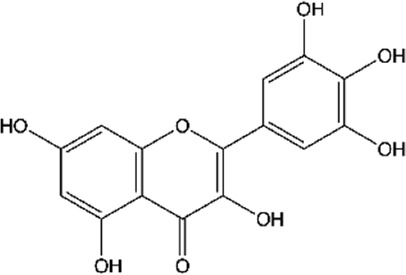	Arunachalam et al. (2019); Lu et al. (2018); Oliveira et al. (2015)
07	Naringenin	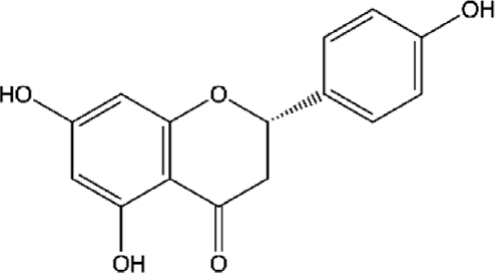	Bisignano et al. (2013); Lu et al. (2018); Oliveira et al. (2015); Zengin et al. (2018)
08	Naringin	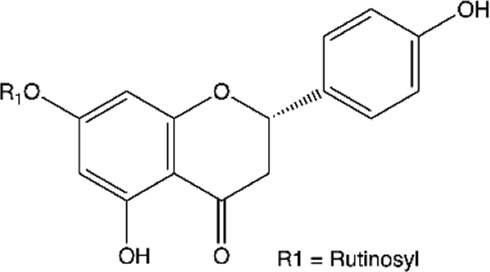	Lu et al. (2018); Oliveira et al. (2015); Zengin et al. (2018)
09	Quercetin	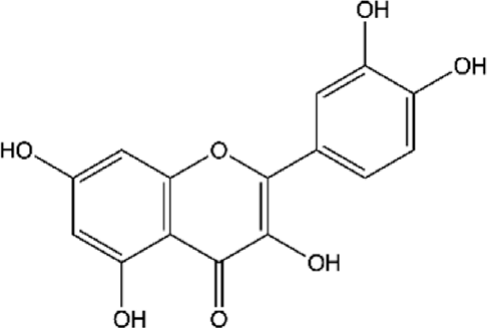	Adzu et al. (2015); Ankolekar et al. (2011); Borges et al. (2020); de Cássia dos Santos et al. (2019); Egas et al. (2018); Ma et al. (2020); Oliveira et al. (2015); Santos et al. (2012); Wang et al. (2018); Zengin et al. (2018)
10	Hyperoside	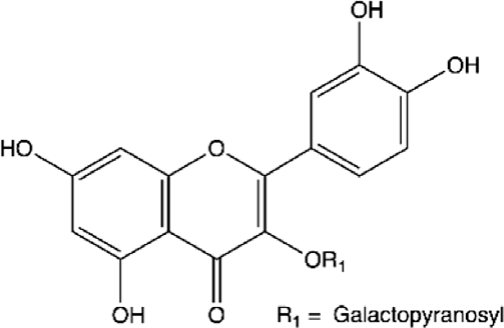	Moon et al. (2011); Santos et al. (2012)
11	Rutin	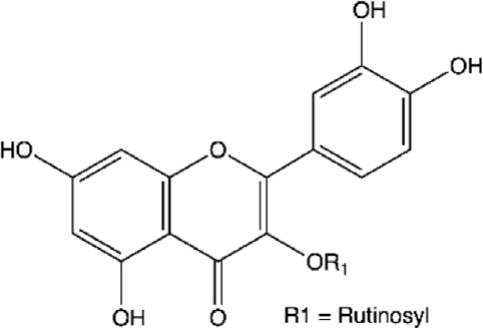	Adzu et al. (2015); Arunachalam et al. (2019); Garro et al. (2015); Lu et al. (2018); Oliveira et al. (2015); Zengin et al. (2018)

The tests for determination of antimicrobial activity included the evaluation of minimum inhibitory concentration (MIC) (Adzu et al., 2015; Arunachalam et al., 2019; Asha et al., 2013; Bisignano et al., 2013; Borges et al., 2020; Cardoso et al., 2018; Cesa et al., 2019; da Silva Junior et al., 2016; de Almeida et al., 2019; de Cássia dos Santos et al., 2019; Egas et al., 2018; Escobedo Hinojosa et al., 2014; Espinosa-Rivero et al., 2015; Fahmy et al., 2020; Garro et al., 2015; Gomez-Chang et al., 2018; Hamad et al., 2015; Ibrahim et al., 2018; Klein-Júnior et al., 2013; Lawal et al., 2014; Moon et al., 2011; Mota Da Silva et al., 2015; Okeleye et al., 2011; Oliveira et al., 2015; Palacios-Espinosa et al., 2014; Park et al., 2016; Pastene et al., 2014; Ribeiro et al., 2013; Santos et al., 2012; Saravanakumar et al., 2019; Spósito et al., 2019; Wang et al., 2018; Zengin et al., 2018), minimum bactericidal concentration (MBC) (Ribeiro et al., 2013; Abreu Miranda et al., 2015; Oliveira et al., 2015; Zengin et al., 2018; Borges et al., 2020), disk diffusion (Brown and Jiang, 2013; Manayi et al., 2013; Minozzo et al., 2016; Lu et al., 2018; Das et al., 2020), well agar diffusion (Mazzolin et al., 2010; Okeleye et al., 2011), agar diffusion (Ankolekar et al., 2011; Marcial et al., 2014), rate of kill (Okeleye et al., 2011), and *H. pylori*-associated gastritis (HAG) *in vivo* model (mice) (Ma X. et al., 2020).

## Discussion

The use of natural products and synthetic variations of their structures is the primary source of novel chemical entities approved as drugs by federal regulatory agencies. Despite the significant advance in combinatorial chemistry, discovering new active compounds through exclusively synthetic routes does not fulfill the role of presenting itself as a primary source of therapeutic innovation. In silico analysis has been used as an optimization tool to identify natural compounds as a valuable alternative to the pharmaceutical industry (Newman and Cragg, 2020).

Natural products and their derivatives represent more than a third of all newly discovered molecular entities approved by the FDA (Food and Drugs Administration). Notably, about 25% are of plant origin (Patridge et al., 2016). Sixty-four percent of these compounds have been used to treat neoplastic diseases. For example, of the 126 drugs discovered between 1981 and 2019, 78 (48%) are natural products for antibacterials.

### Flavonoids

The pharmacological potential of medicinal plants is given by the chemical structures produced by secondary plant metabolism. It presents several biosynthesis mechanisms capable of supplying substances with complex chemical structures. In addition, these structures are responsible for specialized intrinsic functions, favoring the activity in biological environments. They generally possess pharmacophoric regions, which are intricate to create or reproduce through organic synthesis (Mendes et al., 2012).

Flavonoids are among the main classes of secondary metabolites with pharmacological relevance (Wang et al., 2019). These phenolic compounds act on plants as adaptive agents, playing a crucial role in the survival of species against environmental stresses and in response to invasions by microorganisms. This natural function of flavonoids explains the growing interest in studying these compounds in searching for new drugs with antimicrobial activity, mainly due to the lack of effective therapies in the current clinical scenario (Biharee et al., 2020).

**FIGURE 2:**
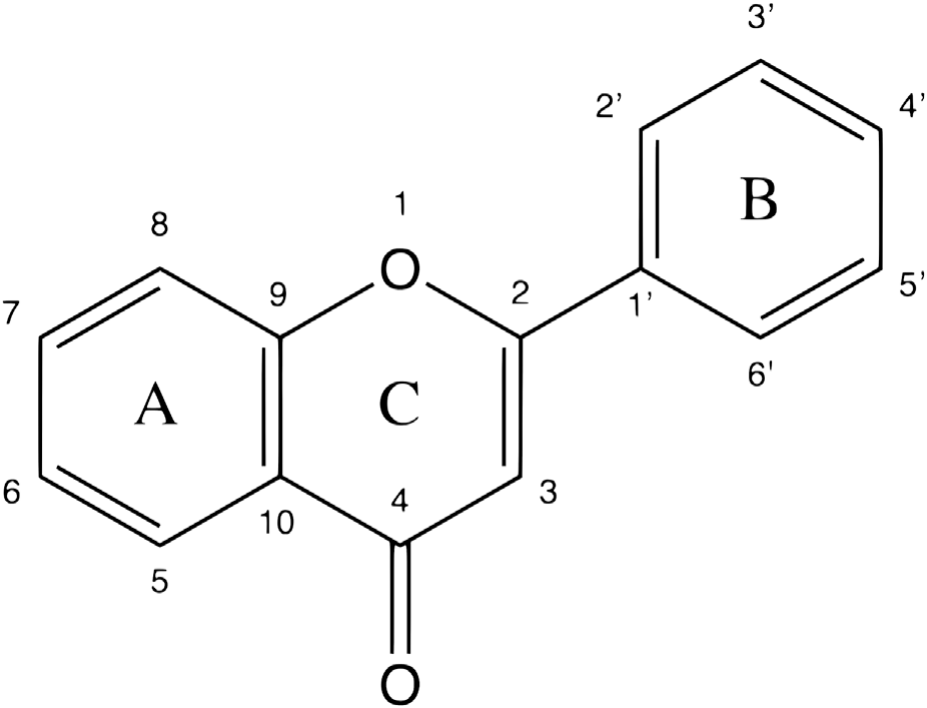
A basic chemical skeleton of flavonoids. General structural representation of a flavonoid, characteristic of 15 carbon atoms in its basic skeleton, corresponding to two benzene rings (A and B) linked by a pyran ring (C).

Moreover, flavonoids represent one of the most important and diversified phenolic groups among natural metabolites. Its occurrence is often associated with the color of plants, and it is frequently found in flowers, fruits, leaves, stems, and seeds (Zuanazzi and Montanha, 2007). The word “Flavonoid” derives from the Latin “Flavus,” which means light yellow. However, the color of the flavonoid often varies according to the species. The basic skeleton of the flavonoids (Figure 2) has a tricyclic structure with 15 carbon atoms, with a chromatic ring (A) fused to a pinane ring (C) connected to an aromatic ring (B), leading to the subcategories of flavonoids (Zuanazzi and Montanha, 2007; Batra and Sharma, 2013; Biharee et al., 2020).

Literature reports have shown that flavonoids play a role in plant survival by preventing the spread of fungal and bacterial pathogens (Cho and Lee, 2015; Piasecka et al., 2015). Furthermore, flavonoid biosynthesis is closely related to defense responses in plant tissues. Significantly, it interferes in the vascular strands of leaves, which are most exposed and susceptible to contamination (Beck and Stengel, 2016). These findings support the hypothesis that flavonoids are potential antimicrobial agents that may be effective against human pathogens.

Due to the great diversity of compounds within the flavonoid group, the highly concentrated extracts exhibit more diverse mechanisms of antimicrobial activity. In other words, they target more components and functions of bacterial cells than other plant secondary metabolites (Cushnie and Lamb, 2005; Górniak et al., 2019). Other advantages of flavonoid-rich extracts with antimicrobial action compared to others are summarized in Table 3.

**TABLE 3:** Advantages of flavonoid-rich extracts with antimicrobial activities compared to other extracts.

Advantages	References
Diverse microbial cell targets	Baker, (2020)
Different mechanisms of conventional antimicrobial drugs	Pandey and Kumar, (2013)
Modulation of antimicrobial resistance mechanisms	Górniak et al. (2019)
Greater possibility of synergistic association with traditional antimicrobials	Cushnie and Lamb, (2011)

#### Catechin and Epicatechin

Catechins, such as catechin and epicatechin, are flavonoids in plants, fruits (e.g., apple, strawberry, kiwi), black and green tea, red wine, beer, chocolate, and cocoa, among others (Grzesik et al., 2018). For example, green tea has a high catechin concentration (Botten et al., 2015), about 1 g/ml in a teacup (Rahardiyan, 2019). Approximately 5–7% of this concentration is epicatechin (Braicu et al., 2013).

Catechins’ antimicrobial activity is related to the interaction of this compound with the cell wall and inner membrane of bacteria and hydrogen peroxide production. One of the proposed mechanisms of action is related to the formation of high molecular mass complexes between this compound and proteins on the surface of the bacterial cell wall. It interrupts substrate transit between the intra and extracellular environment, inhibiting bacterial cell activity (Nakayama et al., 2013).

Some studies report catechin’s antimicrobial activity against gram-positive and gram-negative bacteria. It includes species like *Escherichia coli* (*E. coli*) (Bernal-Mercado et al., 2018), *H. pylori* (Díaz-Gómez et al., 2013), *Staphylococcus aureus* (*S. aureus*) (Martins et al., 2011; Sinsinwar and Vadivel, 2020), and *Bacillus subtilis* (*B. subtilis*) (Fathima and Rao, 2016).

#### Kaempferol

Kaempferol is a natural flavonol present in many edible plants (e.g., broccoli, cabbage, beans, tomatoes, and strawberries) as well as in traditional medicine (e.g., *Ginkgo biloba* L. (Ginkgoaceae); *Moringa oleífera* Lamarck (Moringaceae) (Saldanha et al., 2019). This compound and its glycosides possess several pharmacological properties, such as antioxidant, anti-inflammatory, antidiabetic, anticancer, cardioprotective, neuroprotective, anti-steroidal, anxiolytic, estrogenic/anti-estrogenic, analgesic, anti-allergic and antimicrobial (Mbaveng et al., 2014; Shields, 2017).

Kaempferol’s antimicrobial activity may be associated with its ability to form complexes with the bacterial cell wall, which causes the inhibition of microbial growth (Tatsimo et al., 2012). In addition, the compound blocked the formation of *S. aureus* biofilm in the initial adhesion stage (Ming et al., 2017). This inhibition probably occurs due to the inhibition of enzymes responsible for the beginning of biofilm formation and promoting suppression of the expression of genes of some surface proteins involved in adhesion.

In addition to *S. aureus*, kaempferol and its glycosides have reported activity against *H. pylori* (Escandón et al., 2016), *E. coli* (Wu et al., 2013), *Pseudomonas aeruginosa* (*P. aerugin*osa) (Tatsimo et al., 2012), *Vibrio parahaemolyticus* (*V. parahaemolyticus*), *Bacillus cereus* (*B. cereus*), *Bacillus licheniformis* (*B. licheniformis*) (Sivasothy et al., 2013), and *Enterococcus faecalis* (*E. faecalis*) (del Valle et al., 2016).

#### Luteolin

Luteolin is a flavone naturally found in its glycosylated structure in many edible plant species (e.g., carrot, pepper, peppermint, and oregano) (Omar, 2017). Its pharmacological activities include antioxidant, anti-inflammatory, neuroprotective, anticancer, antidiabetic, and antimicrobial (Dong et al., 2017; Shukla et al., 2019).

Luteolin demonstrates antimicrobial activity against the uropathogenic *E. coli* (UPEC) strain (fei Shen et al., 2014). The compound reduces UPEC adhesion to urinary epithelium cells by decreasing the expression of adhesion proteins in the microorganism’s fimbriae. Furthermore, Luteolin reduced the expression of adhesion-related genes and increased the hydrophilicity, inhibiting biofilm formation.

Other studies also describe the activities of luteolin against *H. pylor*i (Tran Trung et al., 2020), *S. aureus* (Qiu et al., 2011; Joung et al., 2016; Liu et al., 2020), *Listeria monocytogenes* (*L. monocytogenes*) (Qian et al., 2020), *P. aeruginosa*, *B. cereus,* and *Salmonella typhimurium* (*S. typhimurium*) (Rashed et al., 2014).

#### Morin

Morin is a flavone in many fruits and plants of the Moraceae and Myrtaceae families, such as *Maclura pomifera* (Rafinesque) C. K. Schneider (Moraceae); *Maclura tinctoria* L. D. Don ex Steudel. (Moraceae); *Psidium guajava* L. (Myrtaceae); and *Morus alba* L. (Moraceae) (Mbaveng et al., 2014; Shivashankara et al., 2015; Baliga et al., 2019). This flavonoid is attributed to antioxidant, anti-inflammatory, antidiabetic, antihistamine, antitumor, antihypertensive, antiuricemic, neuroprotective, antiviral, and antimicrobial activities (Al-Numair et al., 2014; Caselli et al., 2016).

The antimicrobial activity of morin was demonstrated against *Listeria monocytogenes* can be explained by two mechanisms (Sivaranjani et al., 2016). Firstly, it reduces biofilm formation by inhibiting microbial motility and adhesion and compromising cell-surface and cell-cell interactions. The second mechanism is the interruption of listeriolysin O secretion. It reduces the pathogenicity of *L. monocytogenes* in epithelial cells and macrophages.

Morin acts against *S. aureus* (Amin et al., 2015), *B. cereus*, *Salmonella enteritidis* (*S. enteritidis*) (Arima and Danno, 2002), *E. coli* (Kopacz et al., 2016), and *H. pylori* (Tombola et al., 2003).

#### Myricetin

Myricetin is a flavone encountered in many fruits, vegetables, teas, berries, and red wine (Omar, 2017). Biological activities attributed to this flavone include hypoglycemic (Eddouks et al., 2014), anti-inflammatory (Shukla et al., 2019), anticarcinogenic, and antiviral (Dormán et al., 2016), and antimicrobial (Puupponen-Pimiä et al., 2001).

This compound reduces the expression of genes that encode some virulence factors responsible for bacterial colonization. Moreover, it inactivates host defenses, tissue damage, and nutrient uptake genes in pathogenic strains of *Porphyromonas gingivalis* (*P. gingivalis*) (Grenier et al., 2015). Moreover, myricetin activity was demonstrated against *E. coli* (Puupponen-Pimiä et al., 2001), *S. aureus*, and *Proteus vulgaris* (*P. vulgaris*) (Mori et al., 1987), and *H. pylori* (Tran Trung et al., 2020).

#### Naringenin and Naringin

The flavanone naringenin and its glycoside (naringin) are abundant in the peels of citrus fruits, mainly grapefruit and orange (Jadeja and Devkar, 2013). They present antioxidant, antidiabetic (Srinivasan et al., 2019), anti-inflammatory (Shukla et al., 2019), hypolipemic, antihypertensive, and antifibrotic (Casas-Grajales and Muriel, 2017), and antimicrobial (Céliz et al., 2011) activities.

Naringenin and naringin act against *Salmonella enteritidis* (Yin et al., 2012). The study reported the synergism of these grapefruit juice components with the acidic pH generated. In combination, these factors reduced the adhesion of *S. enteritidis* by inhibiting the bacterium’s acid tolerating response mechanism.

Moreover, other studies reported the activity of naringenin and naringin against *E. coli*, *P. aeruginosa* (Adamczak et al., 2019), *Proteus mirabilis* (*P. mirabilis*), *Acinetobacter baumannii* (*A. baumannii*), *Klebsiella pneumonia* (*K. pneumoniae*), *S. aureus*, *B. subtilis*, *E. faecalis* (Özçelik et al., 2011), and *H. pylori* (Tran Trung et al., 2020).

#### Quercetin

Quercetin is a bioflavonoid obtained from various plant sources (e.g., apple, onion, citrus fruits, and vegetables) (Shankar et al., 2015; Horwitz, 2018). It has several biological activities, such as antioxidant, anti-inflammatory, anticancer (Ay et al., 2016), antiviral (Sathya and Devi, 2017), and antimicrobial (Jaisinghani, 2017).

Feasible antimicrobial mechanisms of action of quercetin have been described (Adeyemi et al., 2020) in *S. aureus* and *E. coli*. Quercetin can initiate the peroxidation of the outer lipid membrane in gram-negative bacteria, such as *E. coli*. It compromises the integrity of the bacterial cell barrier, leading to cell lysis. Additionally, in gram-positive bacteria, quercetin causes oxidative stress and activates the kynurenine pathway. It depletes L-tryptophan reserves, leading to a reduction in bacterial growth.

Studies also report quercetin activity against *P. aeruginosa*, *P. vulgaris* (Jaisinghani, 2017), *P. mirabilis*, *A. baumannii*, *K. pneumoniae*, *E. faecalis*, *B. subtilis* (Özçelik et al., 2011) and *H. pylori* (Brown and Jiang, 2013).

#### Hyperoside and Rutin

Hyperoside (quercetin-3′-O-β-D-galactopyranoside) and rutin (quercetin-3-O-rutinoside) are quercetin glycosides found in vegetables, citrus fruits, and berries. They exhibit several biological activities, such as anti-inflammatory, antithrombotic, antidiabetic, hepatoprotective, antioxidant, antihistamine, antitumor, antiplatelet, antihypertensive, antispasmodic, antiprotozoal, and antimicrobial (Patel and Patel, 2019; Shukla et al., 2019).

Rutin can inhibit biofilm formation in *E. coli* and *S. aureus* (Al-Shabib et al., 2017). The probable mechanism of this activity is related to reducing the production of exopolysaccharides. These molecules are responsible for biofilms’ higher resistance to antimicrobials than planktonic cultures and protect the biofilm by forming multiple layers on its surface that aid in adhesion.

Additionally, hyperoside and rutin activities against *P. aeruginosa* (Sun et al., 2017; Adamczak et al., 2019), *Serratia marcescens* (*S. marcescens*) (Vaquero et al., 2007), *E. faecalis* (Jesus et al., 2018), *Actinomyces viscosus* (*A. viscosus*), *Actinomyces naeslundii* (*A. naeslundii*) (Gutiérrez-Venegas et al., 2019), *S. pyogenes* (Van Der Watt and Pretorius, 2001) and *H. pylori* (Jeong, 2009) are also described.

### Use of Extracts Containing Flavonoids Against *H. pylori* and Its Advantages

**FIGURE 3:**
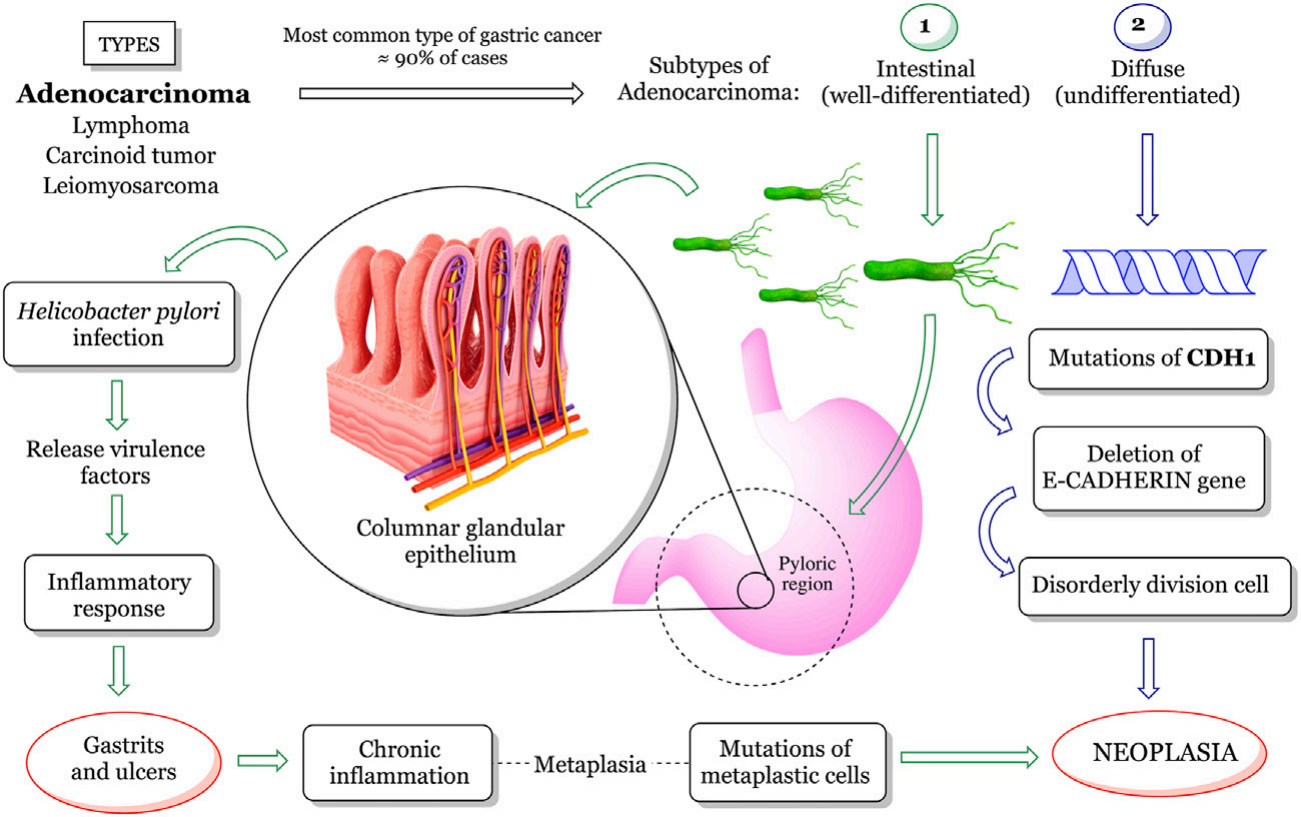
General overview of *H. pylori*-associated gastric cancer development. According to its topology, gastric cancer can be classified into adenocarcinoma, lymphoma, carcinoid tumor, and leiomyosarcoma. Adenocarcinoma is the type of gastric cancer most affecting the population, subdivided into (1) intestinal and (2) diffuse. Type (1) is mainly elicited by an acute immune response induced by *H. pylori* infection. The silent persistence of the bacteria provides a picture of chronic inflammation, consequently inducing gastritis and ulcers that can lead to gastric perforation. As a result, epithelial tissue undergoes metaplasia (cell differentiation) and behaves like intestinal cells, losing function. Metaplastic cells begin a process of disordered division by undergoing gene mutation that ends up in the formation of malignant neoplastic tissue. Type (2) adenocarcinoma is caused by genetic factors that affect the expression of the intercellular adhesion proteins. For example, E-cadherin is responsible for keeping gastric epithelial cells interconnected and controls the cell cycle.

Defined as a group I carcinogen since 1994 by the International Agency for Research on Cancer, *H. pylori* represents about 5% of the total burden of all cancers worldwide (WHO, 2010; Moss, 2017). In addition, *H. pylori* is associated with diseases such as chronic gastritis, peptic ulcer, and gastric mucosa-associated lymphoid tissue lymphoma. This bacterium has a urease enzyme capable of converting the urea present in gastric acid into ammonia, increasing the stomach pH, thus allowing its colonization. Its silent permanence results in chronic inflammation and, consequently, in the appearance of gastritis and ulcers that can lead to gastric perforation. Thereupon, the epithelial tissue begins to undergo metaplasia. The metaplastic cells start the process of uncontrolled division, which undergoes gene mutation and culminate in the formation of malignant neoplastic tissue (Figure 3) (Ladeira et al., 2003; Minozzo et al., 2016).

Among the current treatment strategies for patients with *H. pylori*-associated gastritis and peptic ulcer disease, triple therapy is based on combinations of multiple agents, including bismuth subsalicylate, proton pump inhibitors, H2 blockers, and antibiotics, mainly clarithromycin. Additionally, the eradication of *H. pylori* is indicated for treating lymphoma. Also, other regimens used as adjunctive therapy include probiotics, bovine lactoferrin, and curcumin (Asha et al., 2013).

As expected, the application of multiple drugs is associated with several side effects, making it impossible for the patient to adhere to and abandon the course of treatment. In addition, these interferents can lead to the emergence of resistant strains (Ustün et al., 2006).

**FIGURE 4:**
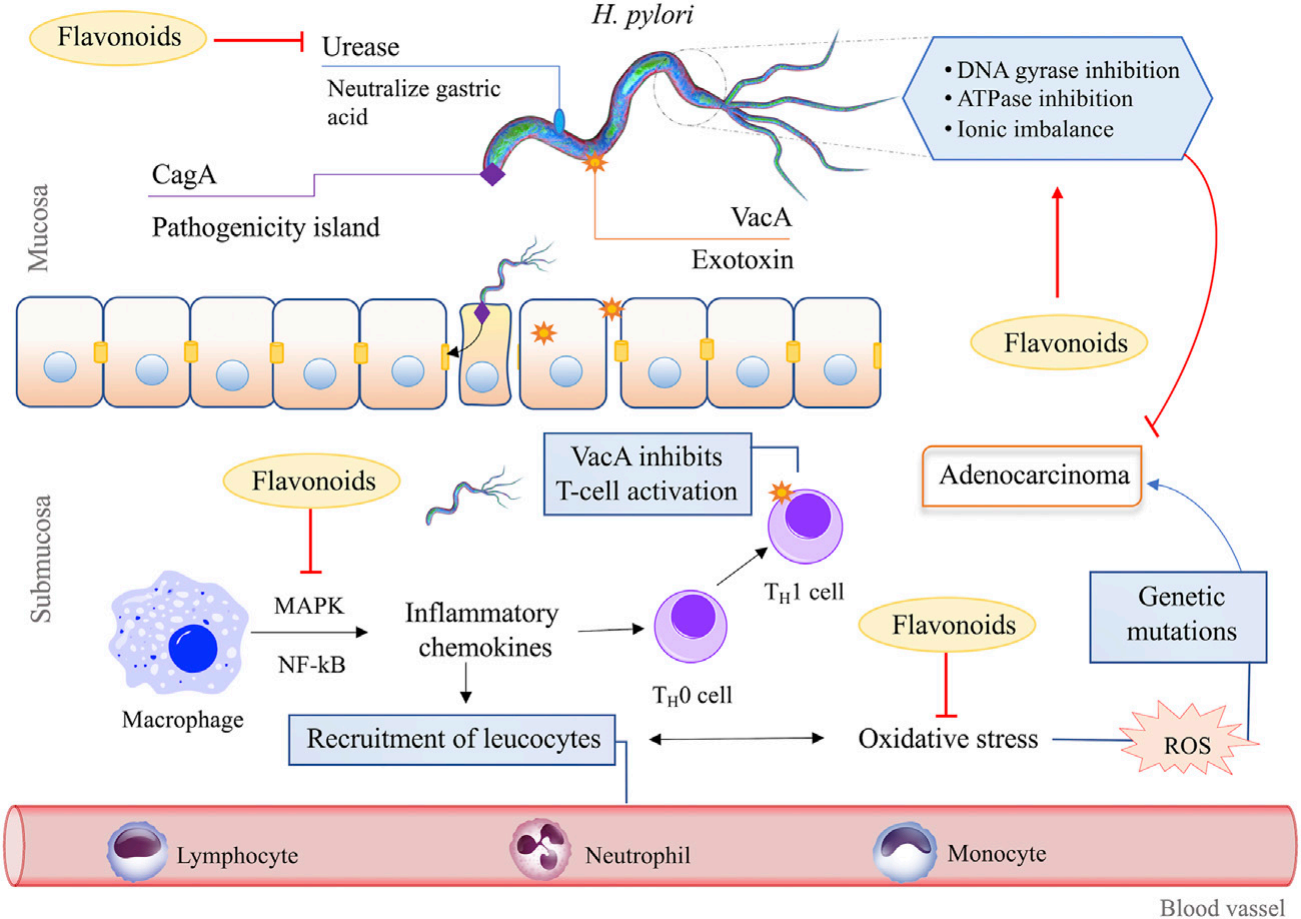
Mechanisms of action of flavonoids against H. pylori. The pathogenesis of *H. pylori* depends on several virulence factors, including urease. This enzyme neutralizes the acidic pH of the gastric medium and facilitates bacteria adhesion to the epithelium. Flavonoids can block this process once they inhibit urease activity. Flavonoids also act by inhibiting enzymes crucial for the reproduction and survival of *H. pylori*, such as DNA gyrase and ATPase. In addition, it induces ionic and metabolic imbalances at the cytoplasmic level, causing bacterial cell wall disruption, which leads to cell death or latency. Once adhered to the gastric epithelium, *H. pylori* induces the release of proinflammatory cytokines and the generation of specific genetic mutations which promote cell apoptosis. Flavonoids have anti-inflammatory activities. They inhibit MAPK and NF-kB pathways and regulate the oxidative stress response in phagocytes and other cells. In addition, some flavonoids act as antioxidant agents, scavenging free radicals and reestablishing the ionic balance. The Establishment of adenocarcinoma from successive gene mutations caused by chronic inflammation and the inhibition of any stage of this process can attenuate, delay or prevent the onset of gastric cancer.

Therefore, the search for new anti-*H. pylori* therapy alternatives promoted the exploration in the field of medicinal plants. As a result, several studies have been conducted, and many natural products have anti-*H. pylori* mechanisms of action proven, such as urease inhibition, DNA damage, protein synthesis inhibition, and anti-inflammatory effects. Moreover, they inhibit some enzymes, such as dihydrofolate reductase and myeloperoxidase N-acetyltransferase (Figure 4) (Baker, 2020).

Preparations containing physiologically active constituents in folk medicine support the therapeutic use of flavonoids. Several pharmacological actions include antimicrobial, antitumor, anti-inflammatory, and antioxidant (Asha et al., 2013). For example, flavonoids are effective against different microorganisms by different mechanisms of action. They cause membrane disruption, antibiofilm action, cell envelope synthesis, nucleic acid synthesis, electron transport chain and ATP synthesis, formation of flavonoid-metal complexes, inhibition of bacterial toxins, and others (Górniak et al., 2019).

As mentioned above, pathogenic bacteria may resist antibiotic drugs through different mechanisms. For example, mechanisms might be the prevention of interaction of the drug with the target, efflux of the antibiotic from the cell, and direct destruction or modification of the drug compound. Moreover, bacteria can interchange resistance genes with surrounding colonies. For example, the β-lactamase gene encodes an enzyme that hydrolyzes the amide bond in the β-lactam ring through transformation (incorporation of naked DNA), transduction (phage-mediated), and conjugation. Gram-negative bacteria interfere with β-lactam ring hydrolysis, whereas Gram-positive bacteria modify the target site of antibiotics (Bush and Fisher, 2011; Bush, 2013).

Occasionally the resistance to antimicrobial agents can be obtained *via* combined mechanisms. For instance, although gentamicin resistance does not rely on antibiotic modification, it is executed by altering the membrane potential and efflux and 16S rRNA methylation (Waglechner and Wright, 2017).

*Cistus laurifolius* L. (Cistaceae) extract has proved anti-*H. pylori*, including against resistant strains (Ustün et al., 2006). Moreover, the extract of *Glycyrrhiza glabra* L. (Fabaceae) maintained its action against *H. pylori* even after being clinically used without any drug resistance (Fukai et al., 2002). Such findings infer how flavonoids may be helpful as lead compounds in the development of a new class of anti-*H. pylori* regimens.

### Possible Mechanisms of Action of Flavonoids Against *H. pylori*

The urease enzyme is considered a potent *H. pylori* virulence factor. It comprises 6% of the synthesized proteins, representing a significant energy investment in colonization. Moreover, its inhibition hinders the survival of *H. pylori* in the gastric environment, which becomes hostile due to high acidity (Ladeira et al., 2003). *In vitro* experiments demonstrated that the flavonoid quercetin, present in *Heterotheca inuloides* Cassini (Asteraceae) extract, promotes high enzyme inhibition (IC_50_ = 132.4 μg/ml) (Egas et al., 2018). Additionally, a complimentary *in silico* study of molecular docking has identified that quercetin interacts with the catalytic site, forming ionic bonds with the zinc cation. Ionic bonds are among the strongest, supporting and inducing high enzyme inhibition activity. Catechin and epicatechin isolated from the *Peumus boldus* extract inhibit urease activity, with IC_50_ values of 66 and 112 µg GAE/ml, respectively (Pastene et al., 2014). However, due to molecules’ sizes and high polarity, they seem to neutralize only the most external pool of urease. It means they could not inhibit the urease cytoplasmic pool, suggesting a more preventive application.

Glabridin and glabrol present in the extract of *Glycyrrhiza glabra* L. (GutGard®) inhibit the dihydrofolate reductase (DHFR) (Asha et al., 2013). DHFR consists of a ubiquitous enzyme in every eukaryotic and prokaryotic cell that plays a crucial role in the synthesis of thymidine. It catalyzes the reduction of 7,8-dihydrofolate to 5,6,7,8-tetrahydrofolate, using NADPH as a cofactor. This reaction is an essential step in the biosynthesis of DNA nucleotide bases and, therefore, plays an essential role in bacteria survival, such as *H. pylori* in the human body. It is interesting to note that in addition to the anti-*H. pylori* activity, GutGard^®^ also has anti-inflammatory (Chandrasekaran et al., 2011) and antioxidant (Mukherjee et al., 2010).

The potential activity of the luteolin-rich extract from *Plectranthus barbatus* (Andrews) Benth. ex G.Don (Lamiaceae) has been proved (Borges et al., 2020). The subinhibitory dose of ethyl acetate (EAF) fraction (128 μg/ml) produced similar morphological changes as did the subinhibitory dose of amoxicillin (0.25 μg/ml). The filamentous cells found and the production of protrusions indicate a possible action on the bacterial cell wall. These data suggest a possible action on PBPs (Penicillin-Binding Proteins), which are involved in the cell septation process, especially the 63 kDa PBP (PBP63). Besides, observations in producing protrusions and blebs suggest a possible action on other PBPs involved in the peptidoglycan wall.

When assessing the intracellular accumulation of quercetin from *Vitis rotundifolia* Michaux (Vitaceae), it was observed a behavior possibly interspersed in the hydrophobic region of the lipid bilayers of the cell envelope, passively diffused through the cell membrane into the cytosol (Brown and Jiang, 2013). It may be an interaction with cell membrane proteins or active import into the cytosol, leading to metabolic imbalance, followed by cell death.

Catechin, epicatechin, epigallocatechin, and quercetin flavonoids in white, green, oolong, and black teas deplete membrane electrons responsible for the transport chain (Ankolekar et al., 2011). They disrupt oxidative phosphorylation and inhibit the proton efflux linked to dehydrogenase. Thereby, they interfere with the flow of electrons at the cytochrome level. However, low hydrophobic and simple soluble phenolic compounds may not be effective. The outer lipopolysaccharide layer of *H. pylori* avoids oxidative phosphorylation. As a result, the membrane creates a hydrophobic microenvironment along the bacterial surface. Also, soluble phenols interrupt the H^+^-adenosine triphosphatase required to synthesize adenosine triphosphate. It causes hyperacidification *via* proton donation in the plasma membrane or in the intracellular cytosolic pathway. Another explanation is that the hydrophobic portion of polyphenols adheres to the cell wall. It would cause destabilization and rupture of the membrane and inhibition of transmembrane transport. These mechanisms can act synergistically: the disturbing and destabilizing effect of polyphenols can make it easier for simple soluble phenols to exercise their hyperacidification.

*Alpinia officinarum* Hance (Zingiberaceae) extracts possess the flavonoids apigenin, galangin, galangin-3-methyl ether, kaempferol, kaempferide, pinobaksin, pinocembrin, quercetin, quercetin-3-methyl ether, and salvagenin (Ma X. et al., 2020). To such metabolites has been attributed the anti-*H. pylori* activity by inhibiting the synthesis of the proinflammatory cytokine interleukin-8 (IL-8) *via* the MAPK pathway, whose gene shows a significant increase in expression in the entire genome of gastric epithelial cells after infection by *H. pylori*. This reduction would result in decreased inflammation and adhesion of the bacteria to the epithelium.

Moreover, the *Lippia integrifolia* (Griseb.) Hieronymus (Verbenaceae) extract demonstrated the flavonoids salvagenin, 6-Hydroxyluteolin 7-hexoside, 6-Methoxyluteolin-hexoside, 6-Methylscutellarein 7-hexoside, B-ring-dimethoxylated Flavone-hexoside and Methoxylated apigenin-hexoside (Marcial et al., 2014). As a result, it exhibited strong antioxidant capacity *in vitro*. Furthermore, it inhibited *H. pylori* adhesion to stomach cells by up to 40%. In comparison, the ethanol-soluble fraction showed up to 60% inhibition rates. Furthermore, the decoction significantly increased the gastric adenocarcinoma cell line (AGS) cell viability at> 10 μg/ml without influencing the proliferation rate. Besides, *H. pylori*-induced IL-8 secretion was significantly reduced by the coincubation of AGS cells with extracts.

### Study Relevance

Here, the activity of flavonoid extracts was investigated against *H. pylori* for gastric cancer prevention and treatment. These studies provided a basis for further investigations that may lead to new clinical trials for proper drug administration. In this perspective, the use of botanical drugs might present as a viable and safe alternative.

Administration of botanic products is a complementary treatment for acute and chronic diseases and preventive care. GutGard^®^ (Asha et al., 2013) is an example currently used to help control indigestion and heartburn and manage *H. pylori* infection at a dosage of 150 mg/day (Ribeiro, 2019). So, using extracts containing flavonoid compounds might be highly relevant to eradicating *H. pylori* as a curative and preventive therapeutic strategy.

Furthermore, botanical drugs are cost-effective compared to synthetic medicines and processed plant derivates (Maqbool et al., 2019). The focus on the herbal products research encourages the investigation of alternative therapeutic modalities for long-standing and persistent health problems.

### Limitations

Studies using pure isolated flavonoids were rare, which required the inclusion of additional extracts in our protocol. Few publications mentioned the mechanism of action of flavonoids on *H. pylori*. Additionally, many articles identified their flavonoids but not their concentration. Furthermore, some studies did not use full botanical taxonomic names, limiting comparative analysis and data translation. Finally, only one *in vivo* study was included in this review, limiting the analytical power of the effectiveness of the flavonoid in the live organism.

## Future Directions

This review on flavonoids’ antimicrobial and anticancer action is in line with recent literature (Al-Ishaq et al., 2021; González et al., 2021; Li et al., 2022). Altogether, data demonstrate the great potential of these compounds in combating *H. pylori* infection and gastric cancer protection. The immense variety of plants that serve as a source of flavonoids can ensure the sustainable production of drugs containing the compounds. In addition, the great potential of these compounds for various health applications is observed. New flavonoids’ extraction methods might allow for better profitability and sustainability. Moreover, dosage control and compound stability offered by smart drug delivery systems can further expand the understanding of the activities of flavonoid-rich extracts against various pathogens (de Lima Nascimento et al., 2019; Gondim et al., 2019).

Furthermore, a broader understanding of the interactions of flavonoids with drugs of choice for the treatment of *H. pylori* may help determine the use of the compounds as adjuvant therapy in the fight against the development of gastric cancer. Also, it is imperative to evaluate the effect of flavonoids on the human microbiome and how the metabolic process of the colonizing microbes might interfere with the activities of the compounds against pathogenic strains. Clinical trials testing the action of these compounds in patients with *H. pylori* infection deserve attention.

## Conclusion

There is no single therapy for *H. pylori* eradication. Instead, an association of an antiulcerogenic drug and two antibiotics with 70–85% success rates. Our data demonstrated the relevance of new studies involving natural products, specifically extracts of plants rich in flavonoids. These compounds have shown promising results in anti-*H. pylori* targeting different mechanisms of action.
